# Identification, characterization and metagenome analysis of oocyte-specific genes organized in clusters in the mouse genome

**DOI:** 10.1186/1471-2164-6-76

**Published:** 2005-05-20

**Authors:** Amélie Paillisson, Sébastien Dadé, Isabelle Callebaut, Martine Bontoux, Rozenn Dalbiès-Tran, Daniel Vaiman, Philippe Monget

**Affiliations:** 1 Physiologie de la Reproduction et des Comportements, UMR 6175 INRA-CNRS-Université François Rabelais de Tours-Haras Nationaux, 37380 Nouzilly, France; 2 Département de Biologie Structurale, LMCP, CNRS, UMR 7590, Universités Paris6 et Paris 7, case 115, 4 place Jussieu, 75252 Paris Cedex 05, France; 3 U709 – INSERM, Pavillon Baudelocque, Hôpital Cochin, 123, Boulevard de Port-Royal, 75014 Paris, and INRA, Département de Génétique animale

## Abstract

**Background:**

Genes specifically expressed in the oocyte play key roles in oogenesis, ovarian folliculogenesis, fertilization and/or early embryonic development. In an attempt to identify novel oocyte-specific genes in the mouse, we have used an *in silico *subtraction methodology, and we have focused our attention on genes that are organized in genomic clusters.

**Results:**

In the present work, five clusters have been studied: a cluster of thirteen genes characterized by an F-box domain localized on chromosome 9, a cluster of six genes related to T-cell leukaemia/lymphoma protein 1 (Tcl1) on chromosome 12, a cluster composed of a SPErm-associated glutamate (E)-Rich (Speer) protein expressed in the oocyte in the vicinity of four unknown genes specifically expressed in the testis on chromosome 14, a cluster composed of the oocyte secreted protein-1 (Oosp-1) gene and two Oosp-related genes on chromosome 19, all three being characterized by a partial N-terminal zona pellucida-like domain, and another small cluster of two genes on chromosome 19 as well, composed of a TWIK-Related spinal cord K+ channel encoding-gene, and an unknown gene predicted *in silico *to be testis-specific. The specificity of expression was confirmed by RT-PCR and in situ hybridization for eight and five of them, respectively. Finally, we showed by comparing all of the isolated and clustered oocyte-specific genes identified so far in the mouse genome, that the oocyte-specific clusters are significantly closer to telomeres than isolated oocyte-specific genes are.

**Conclusion:**

We have studied five clusters of genes specifically expressed in female, some of them being also expressed in male germ-cells. Moreover, contrarily to non-clustered oocyte-specific genes, those that are organized in clusters tend to map near chromosome ends, suggesting that this specific near-telomere position of oocyte-clusters in rodents could constitute an evolutionary advantage. Understanding the biological benefits of such an organization as well as the mechanisms leading to a specific oocyte expression in these clusters now requires further investigation.

## Background

Mammalian oocyte is the only known cell able to activate the zygotic genome after fertilization. Its cytoplasm is also able to reprogram the nucleus of a differentiated cell in cloning experiments. Therefore, it is likely that several genes specifically expressed in the oocytes are responsible for this ability to program diploid genomes. It is the case for the so-called maternal genes such as Maternal Antigen That Embryo Required (MATER), Zygotic Arrest 1 (Zar1), Stella and nucleoplasmin 2 (Npm2) that are all required for normal embryonic development beyond the 1- or 2-cell stage [1,2]. Moreover, new observations have shown that loss of function mutations in the two oocyte-specific genes GDF-9 and BMP-15 are responsible for severe alterations of ovarian folliculogenesis, these alterations being different depending on the hetero- or homozygous state of the individuals in the ovine species [3,4]. So genes specifically expressed in the oocyte seem to play key roles in oogenesis, ovarian folliculogenesis, fertilization and/or early embryonic development. In an attempt to identify novel oocyte-specific genes, several groups have used both mRNA differential display [5] and *in silico *subtraction approaches [6]. By using the latter, we have recently identified six genes of the oogenesin family [7], these genes being present on chromosome 4 in a cluster of almost 1 Mb composed of twelve oogenesin paralogous genes. We have also identified six genes presenting similarities with NALP5/MATER [8] which are specifically expressed in the mouse oocyte, three being clusterized on chromosome 7. We have experimentally validated the specificity of expression for three oogenesin and five NALP genes [7,8]. The fact that these two groups of genes are localized in two clusters indicates that they likely originate from duplications of an ancestral gene.

In the present work, we have used the same *in silico *approach to identify new oocyte-specific genes that are also localized in clusters in the mouse genome. In particular, we have identified three new loci in the mouse genome containing several genes specifically expressed in the oocyte, and two loci containing genes specifically expressed in male and female germ cells, which may thus be called "germ cell loci". We have experimentally verified the specificity of expression for eight of them by RT-PCR and five of them by *in situ *hybridization. Moreover, we have compared the map position of all the "oocyte-clusters" identified so far with that of the other "isolated" oocyte-specific genes. We could show that oocyte-specific clustered genes are significantly closer to telomeres than oocyte-specific isolated genes. Our results emphasize a new vision of gene regulation, where large-scale genome organization likely plays an important function.

## Results

### *In silico *identification of oocyte-specific genes

**Figure 1:**
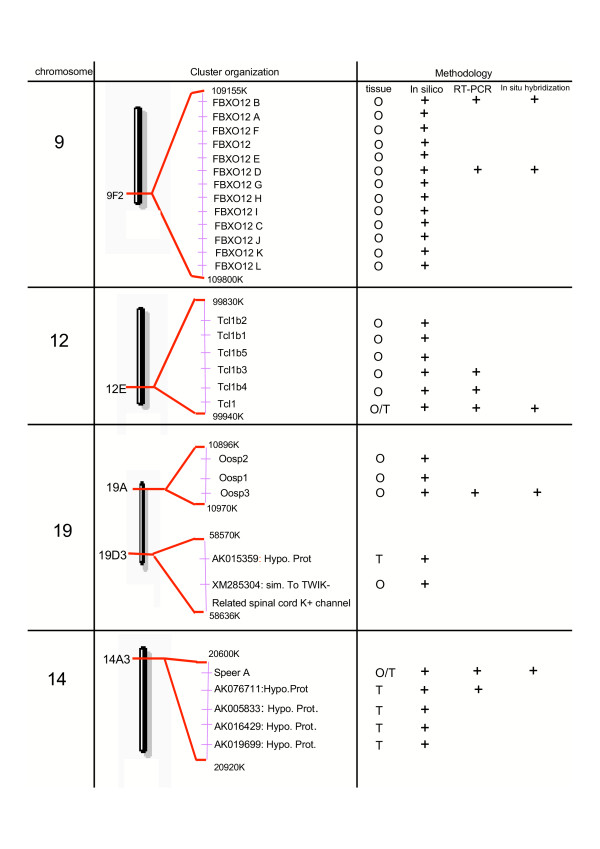
Localization of germ-cell-specific cluster in mouse chromosome 9, 12, 14 and 19 [9]. The presence of mRNAs or ESTs of these different genes in male and female germ cells was assessed by experimental (RT-PCR, Southern blot and in situ hybridization) and/or *in silico *methodologies as described in Methods. O stands for Ovary/Egg; T stands for Testis.

By using the Digital Differential Display (DDD) software, and as previously described [7,8], we found *in silico *a list of approximately 60 genes for which ESTs were exclusively detected in mouse egg libraries. After localization on the mouse genome [9], we noticed that several of these genes were neighbored by genes for which ESTs were exclusively present in egg and/or testis libraries as well. Finally, the number of oocyte-specific genes approached one hundred. Amongst these genes were found most of the well-known oocyte specific genes such as GDF-9, Mater or Zar1, validating the methodology, as well as the 12 oogenesins and the 6 NALP/Mater-like genes [7,8]. In the present work, we have focused our attention towards five other clusters of genes (Fig. 1).

The first cluster is localized on chromosome 9 and is composed of thirteen genes corresponding to the GenBank accession numbers AK054339, AK087710, AK087709, AK087808, XM_284540, XM_486447, XM_486263, XM_488195, XM_356253, AK054298, AK087669, XM_356191 and AK007274. All of these genes encode proteins that contain an N-terminal F-box domain as well as WD40 repeats in the C-terminal region. As the gene corresponding to the GenBank accession number AK087709 is named "F-box only protein 12" (FBXO12), we have renamed the twelve other genes FBXO12A, FBXO12B, FBXO12C, FBXO12D, FBXO12E, FBXO12F, FBXO12G, FBXO12H, FBXO12I, FBXO12J, FBXO12K and FBXO12L (Table 1 & Fig. 1).

**Table 1:** *In silico *informations available on Unigene  about the genes, which are present in the five clusters studied here.

**Name**	**chromosome**	**GenBank**	**cDNA source**
FBXO12A	9 F2	**AK054339**	Ovary
FBXO12B	9 F2	**AK087710**	ovary; in vitro fertilized eggs
			unfertilized egg
FBXO12	9 F2	**AK087709**	ovary, pituitary gland, egg, pre-implantation
			embryo, mid-gestation embryo
FBXO12C	9 F2	**AK087808**	ovary, egg
FBXO12D	9 F2	**XM_284540**	in vitro fertilized eggs ;
			Unfertilized Egg
FBXO12E	9 F2	**XM_486447**	Ovary, Egg, Pre-implantation Embryo
FBXO12F	9 F2	**XM_486263**	Egg, Pre-implantation Embryo
FBXO12G	9 F2	**XM_488195**	Kidney, Egg, Pre-implantation Embryo, Neonate
FBXO12H	9 F2	**XM_356253**	Egg
FBXO12I	9 F2	**AK054298**	Ovary, Egg, Pre-implantation Embryo
FBXO12J	9 F2	**AK087669**	Brain, Ovary, Skin, Egg, Pre-implantation Embryo
FBXO12K	9 F2	**XM_356191**	Ovary, Thymus, Egg, Pre-implantation Embryo,
FBXO12L	9 F2	**AK007274**	Brain, Testis, Pre-implantation Embryo
			
Tcl1b2	12 E	**NM_013775**	Egg, Pre-implantation Embryo
Tcl1b1	12 E	**BC052337**	Egg, Pre-implantation Embryo
Tcl1b5	12 E	**NM_013776**	Pituitary Gland, Egg, Pre-implantation Embryo, Mid-gestation Embryo
Tcl1b3	12 E	**NM_013772**	embryo ; egg
Tcl1b4	12 E	**NM_013774**	embryo ; egg
Tcl1	12 E	**BC052336**	Unfertilized Egg ; in vitro fertilized eggs
			embryo; egg; retina
			
Speer A	14 A3	**XM_138939**	unfertilized egg; in vitro fertilized eggs
			embryo; egg; ovary; round spermatids
	14 A3	**AK076711**	testis
	14 A3	**AK005833**	testis
	14 A3	**AK016429**	testis
	14 A3	**AK019699**	testis
			
Oosp2	19 A	**XM_129237**	Ovary, Pre-implantation Embryo
Oosp1	19 A	**AF420487**	Egg, Pre-implantation Embryo, Mid-Gestation
			embryo
Oosp3	19 A	**XM_129191**	unfertilized egg; in vitro fertilized eggs
			ovary; embryo
			
trik channel	19 D3	**XM_285304**	in vitro fertilized eggs; egg
unknown	19 D3	**AK015359**	testis

The second cluster contains the so-called Tcl1 (T-cell leukemia/lymphoma protein 1) and Tcl1b genes, and maps on chromosome 12. This cluster contains six genes related to Tcl1 gene and is predicted to be oocyte-specific *in silico *(Fig. 1 & Table 1). The protein encoded by this gene shares similarities with the protein encoded by the gene *MTCP1*, also involved in chromosomal translocations in T-cell proliferative disease. Its structure consists of an eight-stranded β-barrel with a particular topology, with the N- and C-terminal halfs being linked by a pseudo dyad [10].

We have identified another cluster on chromosome 14, which was composed of five genes. One of them encoded for a protein that belongs to the Speer (SPErm-associated glutamate (E)-Rich protein) family, characterized by a very high proportion of alpha-helical secondary structure. We have thus renamed this gene SpeerA (Genbank identification number XM_138939). The corresponding Speer proteins are weakly similar to proteins that contain interacting domain with cytoskeletal as well as nuclear matrix proteins such as actin and dynein. *In silico*, ESTs of SpeerA are present in egg libraries but also in round spermatid libraries (Table 1). The ESTs corresponding to the four nearest neighboring genes, corresponding to the GenBank identification numbers AK076711, AK005833, AK016429 and AK019699, have been exclusively detected *in silico *in testis libraries (Fig. 1 & Table 1). The gene corresponding to the AK016429 accession number encoded for a protein that presented no clear similarity with any known protein. The three other genes likely encoded for untranslated RNAs, as no long ORF could be detected in their sequence.

**Figure 2:**
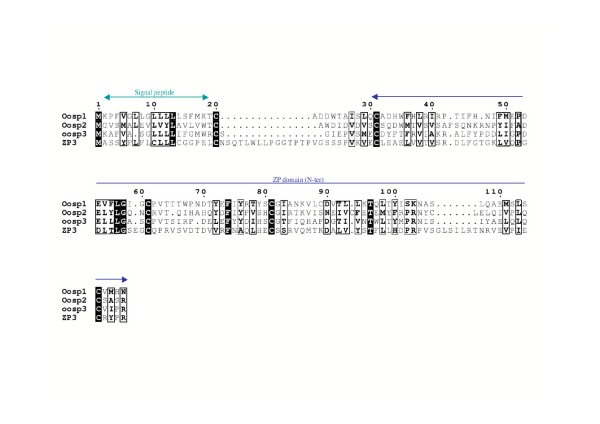
Alignment of Oosp protein sequence with the N-terminal part of the ZP domain (150 amino acids) of ZP3. Identities and similarities are shown with black boxes and black frames, respectively. An hypothetical disulfide bond is suspected between Cys78 and Cys98 according to experimental data obtained on the corresponding Cys61 and Cys81 of ZP3 [11].

Two other clusters map to chromosome 19. The first one is composed of three genes (Genbank identification numbers XM_129237, AF420487, and XM_129191). The corresponding three protein sequences share about 47% of similarity and can be aligned with the 150 first amino acids of domains of the Zona-Pellucida family (Fig. 2). Secondary structure predictions indicate mainly a β-fold for this domain, in which the conserved Cys78 and Cys98 might form a disulfide bond, according to experimental data obtained on the corresponding Cys61 and Cys81 of ZP3 [11]. The C-terminal ends of Oosp proteins (not represented) are highly variable and cannot be aligned with the much more large C-terminal part of ZP domains. The gene corresponding to the GenBank accession number AF420487 is the well-known "oocyte secreted protein 1" (Oosp1) [12]. So we have renamed the two other genes Oosp2 and Oosp3 (Table 1 & Fig 1).

The second cluster on chromosome 19 is composed of an oocyte specific gene, corresponding to the XM_285304 GenBank identification number (Fig. 1), the protein of which is similar to a TWIK-Related spinal cord K+ (Trik) channel. The other gene, corresponding to the AK015359 number, was predicted to be testis-specific *in silico *(Table 1), and encoded for an unknown protein (Fig. 1).

### Expression analysis

**Figure 3:**
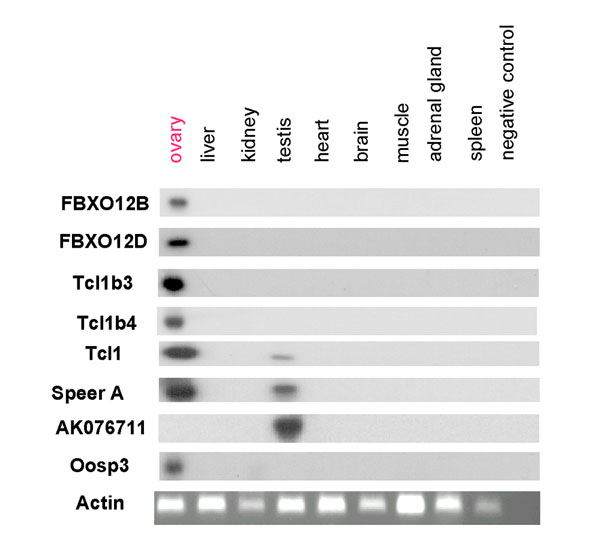
Expression analysis of FBXO12B, FBXO12D, Tcl1b3, Tcl1b4, Tcl1, SpeerA, AK076711, Oosp3 and actin by RT-PCR in mouse tissues. Except for actin, total RNA from different tissues were subjected to Southern blot analysis of the RT-PCR products as described in Methods.

By analysis of RT-PCR products on BET-stained gels, FBXO12B, FBXO12D, Tcl1, Tcl1b3, Tcl1b4, SpeerA and Oosp3 appeared to be exclusively expressed in the ovary, whereas the gene corresponding to the AK076711 GenBank identification number appeared to be exclusively expressed in the testis (data not shown). By contrast, actin was readily amplified in all tissues. To increase the sensitivity of detection of these transcripts, Southern blot experiments were performed on RT-PCR products, confirming these results, except for Tcl1 and SpeerA genes for which a slight expression was also detected in the testis (Fig. 3).

**Figure 4:**
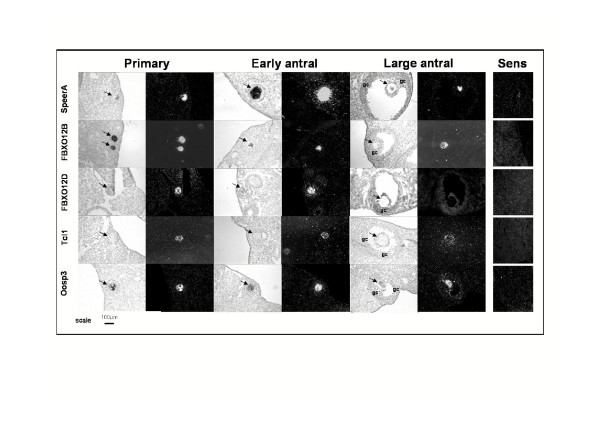
Localization of SpeerA, FBXO12B, FBXO12D, Tcl1 and Oosp3 mRNAs by in situ hybridization. Black arrows point: oocytes; gc: granulosa cells

In situ hybridization confirmed that within the ovary, SpeerA, FBXO12B, FBXO12D, Tcl1 and Oosp3 were exclusively expressed in oocytes (Fig. 4). Transcripts were detected in primary follicles, as well as in early and large antral follicles (Fig. 4), except for FBXO12D and Tcl1, for which transcripts were only slightly if at all detected in large antral follicles (Fig. 4).

### Metagenome analysis

Altogether, the chromosome localization of 92 oocyte-specific genes was known, amongst the ~100 *in silico*-predicted oocyte-specific genes recovered. Amongst these 92 genes, 48 were organized in 7 "oocyte-clusters" (Table 2). We did not consider the two "germ-cell clusters" on chromosomes 14 and 19 in this analysis, but we included the oogenesin and Nalp9 clusters previously described by our group [7,8], as well as the Obox cluster described by Rajkovic et al. (2002). The other 44 oocyte-genes were considered as "isolated" in the mouse genome. For these latter genes, the average distance expressed in relative distance from the chromosome end ranged from 2.5% to 49.8% (average = 24 ± 16%). By contrast, the 48 oocyte-specific genes organized in clusters mapped between 5.6% and 20.6% from the closest chromosome end (average = 12.6 ± 5.3%). A Student *t *test was performed to compare the two categories, considering only one position for each cluster, and resulted in a value of 0.035. Similar analysis was performed taking into account the number of genes before and after the locus/cluster under scrutiny. This analysis gave rise to similar results. Therefore, oocyte-specific genes that are organized in clusters map significantly nearest chromosome ends than those that are "isolated".

**Table 2:** description of the 7 clusters of oocyte-specific genes that were considered for the metagenome analysis: clusters of the oogenesin proteins [7], the Oas proteins [38], the Obox proteins [39], the Nalp9 proteins [8], the FBXO12 proteins, the Tcl1 proteins and the Oosp proteins.

**chromosome**	**name of the cluster**	**number of genes localized in the cluster**
9 E1	Oogenesin	12
5 F	Oas	3
7 A1	Obox	8
7 A3	Nalp9	3
9 F2	FBXO12	13
12 E	Tcl1	6
19A	Oosp	3
	**Total**	48

## Discussion

The present work and two previous identifications of the oogenesin genes [7] and of the mater family genes [8] demonstrate that the *in silico *subtraction is an efficient and reliable methodology to identify new oocyte-specific genes. By localizing these genes on the mouse genome, we have discovered other "germ-cell" specific genes, organized in clusters. RT-PCR as well as *in situ *hybridization experiments confirmed their specificity of expression in the ovary and/or in the testis. As for GDF-9, Mater, Zar1 or Stella, these genes may play important roles in folliculogenesis, fertilization and/or early embryonic development.

Amongst the "oocyte-specific clusters" presented here, the Tcl1/1B has previously been described. Indeed, in a previous work on human TCL1 and TCL1B genes, the six murine Tcl1 and Tcl1b1-5 orthologous genes were already shown to be expressed in oocytes and two-cell embryos but not in other adult tissues except in lymphoid cell lines, where overexpression of Tcl1 in these cells leads to leukemia or lymphoma [13]. Tcl1 was also shown to be expressed in testis, as previously described [14,15], its overexpression inducing the formation of testicular seminomas [16]. Interestingly, Tcl1^-/- ^female mice are sterile, as early embryos are unable to undergo normal cell cleavage up to 8-cell stage, the majority of embryos being even unable to proceed beyond the 4- to 8-cell stage [16]. Therefore, like Mater and Zar1, Tcl1 can be considered as a maternal effect gene as well. It is possible that part of the effects of this gene family is to promote cell survival and/or proliferation by activating the Akt kinase as previously observed [17,18].

The F-box proteins, encoded by the thirteen genes localized on chromosome 9 cluster, might specifically interact with other proteins in the oocyte. Indeed, the F-box motif is present in numerous proteins known to serve as a link between a target protein and an ubiquitin-conjugating enzyme. In particular, it has been shown that the Early Mitotic Inhibitor (Emi)-1, which contains an F-box domain, is able to regulate mitosis progress by inhibiting premature anaphase promoting complex/cyclosome (APC) activation [19], the APC complex being composed of an ubiquitin ligase coupled with the SCF (Skp1-Cullin-F-box protein) complex [20]. So it is possible that the oocyte-specific F-Box proteins would participate in the stabilization of the meiotic process at the prophase I stage.

Concerning the cluster on chromosome 14, in situ hybridization showed a high level of expression of SpeerA gene in the mouse oocyte, corroborating previous results from Matzuk's group [6]. By analyzing the RT-PCR results, we found that this gene was also expressed in the testis, likely in male germ cells. The Speer family has recently been described as a new family of 14 genes. Interestingly, SPEER-1, SPEER-2, and SPEER-4D were recently shown to be expressed specifically in the mouse testis [21]. These genes have open reading frames of approximately 200-220 amino acids, and encode for proteins that exhibit a high proportion of alpha-helical secondary structure, comprising approximately 15% glutamate residues. According to their possible interaction with cytoskeletal proteins such as actin or dynein, it has been hypothesized that the SPEER may be nuclear matrix proteins involved in the reorganization of the spermatocyte/spermatid nucleus [21]. SpeerA, discovered in our study (XM_138939), shares about 34% amino acid sequence similarity with SPEER-4A gene, which could suggest that it plays a similar role in the oocyte. Of note, in contrast to most of the oocyte clusters, the four testis-specific genes neighboring the SpeerA gene were not structurally related to each other.

One of the clusters on chromosome 19 contains the well-known oocyte-specific gene Oosp-1 [12], and two structurally similar genes, these three genes clearly sharing a ZP-like domain. This suggests that these genes encode for structural proteins involved in the formation of certain components of the zona pellucida. A structural analysis of the sequences of these proteins is underway in our laboratory to further investigate the structural specificity of these proteins. The biological roles of the two genes present in the other cluster on chromosome 19, as well as the role of the testis-specific genes present on the chromosome 14 cluster are unknown.

Overall, for all of these genes, further experiments as well as structural analysis are now necessary to investigate their biological roles in oocyte and follicle maturation. Investigating such a role will require functional studies such as identification of partners and/or targeted invalidation.

As we previously discussed, the targeted invalidation of MATER gene leads to female infertility due to a blockage of early embryonic development [22]. This suggests that the oocyte-specific MATER-like genes, i.e. the Nalp9A-F genes that we have identified [8] are not able to compensate the absence of the MATER/Nalp5 product in Mater-/- mice. Similarly, invalidation of Tcl1 gene in the mouse was not clearly compensated by the five oocyte-specific Tcl1B genes. Overall, this suggests that despite an apparent redundancy in the expression of these structurally similar genes, every member of the oocyte Nalp and Tcl1 family of paralogous genes seem to have very specific biological roles in female reproduction. Invalidation of each of these genes is now necessary to study their specific biological role. Moreover, as expected, most oocyte-specific genes organized in clusters are paralogous genes, originating presumably from a common ancestral gene by successive duplication events. It is the case for the oogenesin, Nalp9, Fbxo12, Tcl1 as well as the Osp1-3 genes. It is clearly not the case for the clusters localized on 14A3 and 19D3 (Fig. 1). Nevertheless, such an organization of "oocyte- or germ-cell clusters" suggests the presence of specific genomic regulatory regions in the vicinity of these genes.

As we depicted in figure 1, the SpeerA gene is localized on a cluster on chromosome 14, in the vicinity of four unknown testis-specific genes. On chromosome 19, the oocyte-specific gene related to TWIK-Related spinal cord K+ channel is localized near the gene corresponding to AK015359 GenBank number, which is predicted *in silico *to be testis-specific as well. Interestingly, we have previously shown that in the oogenesin cluster on chromosome 4, the oogenesin-4 gene is also slightly expressed in the testis. Of note, Pramel1 gene, which is localized near oogenesin4, has also been described as a male germ-cell specific genes [7,23]. Similarly, in the oocyte-specific Nalp cluster on chromosome 7, we have shown that Nalp9D is also clearly expressed in the testis, presumably in germ cells. So the notion of oocyte-specific cluster would not be systematically restricted to female germ cells, and would be extended in some cases to "germ-cells cluster" as suggested in this work.

One striking observation of our work is the difference in chromosome localization between oocyte-specific genes organized in clusters and oocyte-genes that are not. If genes were located at random on the chromosome and that the distance to the closest end is measured, then the expected range would be 0% to 50%, and one would expect to reach an average distance of 25% if enough genes are taken into consideration. In our study, isolated genes were very close to this value (24%) whereas clusters were located in average at half of this distance (12%). Gene clusters presumably originate from local gene duplications [24], which are now recognized as efficient motors of diversification in metazoans. Of note, the location of gene families near telomeres has a deep sense in terms of rapid evolution. Indeed, subtelomeric sequences are recognized as the most rapidly evolving regions in the genome, maybe due to the high level of recombination present in these regions [25]. On the other hand, clusters of genes are the entry gates for accumulation of mutations, without the risk of noxious consequences for the organism, and therefore enable the development of new functions, as clearly illustrated by the variants of the human hemoglobin beta chain, which adapts the foetus to successive oxygen environments during development [26,27]. For mammals with a high reproductive potential, such as mice and other rodents, the possibility of rapid evolution for genes involved in ovarian function may be a great advantage. Therefore, one can hypothesize that the specific near telomere position of oocyte-specific gene clusters corresponds to an evolutionary advantage.

Interestingly, the genes that we have described here are strictly silenced in non-ovarian tissues, and one may hypothesize that the specific near telomere position of oocyte clusters would contribute towards such a silencing. In particular, it has been shown that telomeric and pericentric regions of chromosomes are mainly composed of heterochromatin in most eukaryotic genomes. Moreover, euchromatic genes relocated by chromosomal rearrangement or by transposition in the neighbourhood of heterochromatin can be silenced in drosophila [28]. In yeast, a similar phenomenon of reversible gene silencing near telomere has been called Telomere Position Effect (TPE) [29,30]. The presence of TPE has also been observed in human cells [31]. Actually, a very specific nuclear topology would explain such a phenomenon. Indeed in yeast, the 32 telomeres cluster at the nuclear periphery in 8 to 10 groups, organized in compartments rich in histone-binding silencing factors [32]. Testing if the telomeres would be organized in very specific compartments in oocyte and in other tissues requires further investigations.

## Conclusion

We have studied five clusters of genes specifically expressed in female, some of them being also expressed in male germ-cells. Moreover, contrarily to non-clustered oocyte-specific genes, those that are organized in clusters tend to map near chromosome ends, suggesting that this specific near-telomere position of oocyte-clusters could result in an evolutionary advantage. Understanding the biological roles as well as the mechanisms underlying such a specific expression now requires further investigation.

## Methods

### *In silico *identification of oocyte-specific genes

As previously described [7,8] three cDNA libraries derived from mouse unfertilized eggs (dbEST library ID.10029), 2-cell egg (dbEST library ID.5391) and in vitro fertilized eggs (dbEST library ID.2589) were submitted to digital differential display analysis [33] to identify oocyte-specific ESTs that are not found in several non normalized cDNA libraries from different adult somatic non-tumoral tissues (brain, kidney, stomach, liver, lung, spleen, muscle, heart, skin, bone marrow, adipose tissue, adrenal gland). The physical localization of identified genes on the mouse chromosomes was retrieved from [9]. The presence of specific domains within the corresponding proteins was determined on [34] as well by a Blast search of homologous proteins [35]

### RT-PCR analyses

Total RNA was extracted from whole adult tissues (ovary, liver, kidney, testis, heart, brain, muscle, adrenal gland and spleen) using RNAble reagent according to the manufacturer's procedure (Eurobio, Les Ulis, France). Reverse transcription was performed for 1 hour at 42°C in a total volume of 20 μl with 2 μg total RNA per sample following standard procedure. Five μl of the cDNA product was amplified by PCR using primers described in Table 3. RT-PCR products were also analyzed by Southern blot. Briefly, the RT-PCR products were fractionated on 1% agarose gel, transferred to Hybond-N+ membrane (Amersham-Pharmacia) and hybridized with the corresponding cDNA fragment labeled by random priming (1 × 10^6 ^cpm/ml) as described previously [36].

**Table 3:** Primers used for RT-PCR amplification of cDNA fragments of FBXO12B, FBXO12D, Tcl1b3, Tcl1b4, Tcl1, SpeerA, AK076711, Oosp3 and actin.

**Gene**	**Primers**		**annealing temperature**	**fragment size (pb)**
FBXO12B	Upper	ATTTGCCTCGTTTGCCTCTGA	49°C	890 pb
	Lower	GGTTATCCTGTCTTCCCTTCT		
FBXO12D	Upper	AGCCCTGTCCTTTTCCTGTCA	50°C	491 pb
	Lower	AATAGTCTGGTTTCCCCTCAC		
Tcl1b3	Upper	TGGTAGACAGTGGGTAGTTGC	54°C	585 pb
	Lower	CAGGTGGAGGAGTTTGAATGG		
Tcl1b4	Upper	TAAGAAGGCAGCCAACCAGAC	54°C	838 pb
	Lower	CTACCCAGCACCAGGCGAACT		
Tcl1	Upper	TTTTATCACGGACTGGCATTG	54°C	1208 pb
	Lower	CCCTCATTTATTGGCATCTCG		
SpeerA	Upper	TGAAGCAGGAAAATAGGAGA	51°C	900 pb
	Lower	CCAAACAGAAAACCAAACAC		
AK076711	Upper	GGCTCCCAGAGGCTGAATCA	61°C	769 pb
	Lower	TAAGGGTGTCCAAAGAATCA		
Oosp3	Upper	TATTTGGGATGTGGAGATGTT	52°C	416 pb
	Lower	TTGAGGAGGAGGGTGGAAGTC		
Actin	Upper	ACGGAACCACAGTTTATCATC	60°C	180 pb
	Lower	GTCCCAGTCTTCAACTATACC		

PCR products were purified from the agarose using the gel extraction kit QIAEX II (Qiagen, Hilden, Germany) and inserted into pGEM-T vector (Promega, Madison, WI, USA). Identity of the selected clones was checked by sequencing.

### In situ hybridization

Frozen ovaries from 3 female mice in di-estrus, 3 female mice in proestrus and 3 female mice in estrus were serially sectioned (10 μm) with a cryostat to perform in situ hybridization experiments using ^35^S-labeled cRNAs probes as previously described [37]. The specificity of the hybridization signals was assessed by comparing the hybridization of the cRNA antisense with the corresponding sense probes. Histological determination of follicular size and degree of atresia was performed on adjacent sections stained with Feulgen [37].

### Metagenome analysis

For the metagenome analysis, we have taken account for all the oocyte-specific genes identified so far by the *in silico *methodology (data not shown), including well known genes (Mater, Zar1), as well as the 12 genes of the oogenesin family [7], and the 6 genes similar to NALP5/MATER [8] that we have previously identified. A total of 92 genes were analyzed, 44 of which are isolated on the chromosomes while 48 were organized in 7 clusters (Table 2). To study the relative distribution of these two subgroups of genes, their relative distance to the closest chromosome end was calculated, and a global Student t-test was calculated to compare the two subgroups. To avoid any bias due to the fact that genes in clusters often comes from duplication of ancestral genes, bias which would artificially increase their coefficient in such a test, we have considered only one position for each cluster. Moreover, although genes are not strictly homogenously distributed along the chromosomes, the clusterized and the isolated oocytes genes considered for the analysis do cover all the mouse chromosomes, thereby limiting the risk of a bias.

## Authors' contributions

AP and SD performed most of the in silico investigation, as well as the molecular biology and the in situ hybridization experiments. IC contributed to the structural analysis of the Oosp-cluster genes. MB participated to the in situ hybridization experiments and DV performed most of the metagenome analysis of the oocyte-genes organized in clusters. PM was assisted by RD-T to coordinate this work.
